# New National Air-Kerma Standard for Low-Energy Electronic Brachytherapy Sources

**DOI:** 10.6028/jres.119.022

**Published:** 2014-10-06

**Authors:** Stephen M Seltzer, Michelle O’Brien, Michael G Mitch

**Affiliations:** National Institute of Standards and Technology, Gaithersburg, MD 20899

**Keywords:** air kerma, electronic brachytherapy sources, free-air ionization chamber, primary standard, spectrometry, x rays

## Abstract

The new primary standard for low-energy electronic brachytherapy sources for the United States is described. These miniature x-ray tubes are inserted in catheters for interstitial radiation therapy and operate at tube potentials of up to about 50 kV. The standard is based on the realization of the air kerma produced by the x-ray beam at a reference distance in air of 50 cm.

## 1. Introduction

The use of insertable, low-energy, miniature x-ray tubes for interstitial radiation therapy of brain, breast, and other tumors was introduced by Photoelectron Corp. in the mid 1990s [1], work which has been carried onward by Carl Ziess, Ltd., as their INTRABEAM source. Newer products have been developed by Xoft, Inc., since the mid 2000s [2]. The application of these devices has been termed electronic brachytherapy, as it provides an effective alternative to the use of sealed, photon-emitting, radionuclide sources in a number of applications; it is thus similar to traditional sealed-source brachytherapy, but utilizes an electrically powered source. The use of such sources requires basic dosimetry to ensure the delivery of the desired absorbed dose in the target. In the U.S., the dosimetry should be traceable to primary national standards, preferentially those maintained by the National Institute of Standards and Technology (NIST). The purpose of this report is to document the NIST air-kerma measurement standard for these electronic brachytherapy sources, *i.e.*, the Axxent^1^ sources of Xoft, Inc., which operate at tube potentials of 50 kV and anode currents of 300 μA.

The NIST has long maintained measurement standards for x-ray beams produced by accelerating potentials up to 300 kV [3, 4], and has developed measurement standards for low-energy-photon-emitting, sealed brachytherapy sources [5, 6]. These standards are based on the realization of air kerma produced by the sources in question using free-air chambers (FACs). The traditional NIST reference-radiation beam qualities have been developed using well-collimated fixed anodes and highly stable, precision x-ray generators with either W, Rh, or Mo anodes. The air kerma produced by these beams is used for the calibration of measuring instruments. The NIST standards for low-energy-photon-emitting, sealed brachytherapy sources are used to establish the air kerma and then transfer the measurement to (calibrate) well-type ionization chambers, both at the NIST and often at secondary calibration laboratories such as the American Association of Physicists in Medicine’s (AAPM) Accredited Dosimetry Calibration Laboratories. The dissemination of measurement standards for limited-lifetime electronic brachytherapy sources clearly fall into this latter procedure, but suitable facilities for using a NIST standard FAC with these novel sources was lacking. A suitable facility was completed in 2009, and the complete characterization of the NIST FAC for the sources available to us has been developed and is described below. Details of the required development of the FAC’s correction factors are provided. Results of the air-kerma comparison between two primary NIST x-ray standards that validates the corrections are provided, as well as the acceptance of a transfer instrument that can be used by secondary calibration facilities or users of the Axxent sources to provide traceability of the measurement of air kerma.

## 2. The NIST Electronic Brachytherapy Facility

**Fig. 1:**
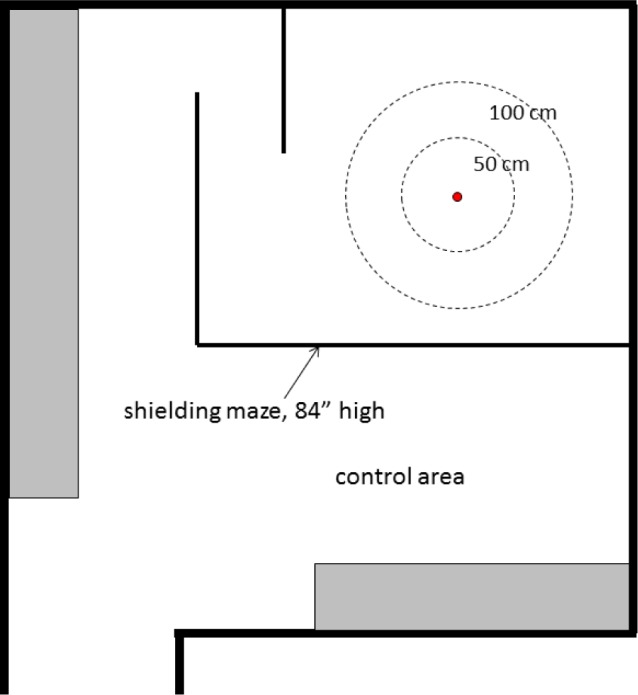
Layout of the NIST electronic brachytherapy dosimetry facility.

**Fig. 2:**
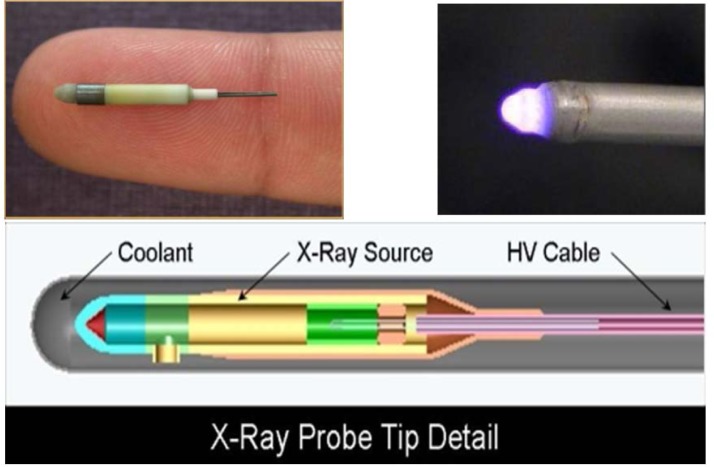
The Xoft Axxent miniature x-ray source.

**Fig. 3:**
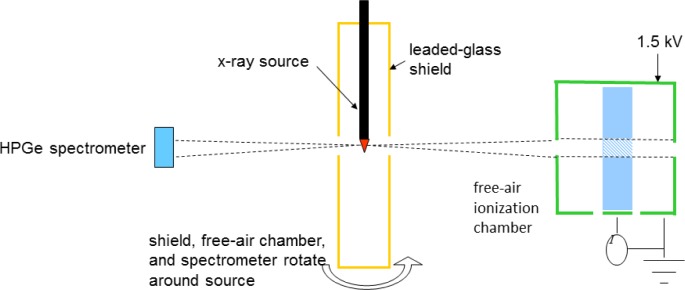
Schematic of original measurement geometry.

Figure 1 shows a schematic overhead view of the measurement room. The design was to initially accommodate the Xoft Axxent miniature x-ray source in its circulated-water cooling catheter, fixed at the center of the bull’s eye in Fig. 1. The walk-in shielding enclosure provides for personnel safety during measurements. The S700 source, shown in Fig. 2, is contained in a water-cooling catheter. The original geometric setup is shown in Fig. 3 where the source is aligned vertically and enclosed in a leaded-glass structure with apertures designed to collimate the x-ray beam, transverse to the source axis, in the directions to the measurement devices. As originally conceived, a motor controls the rotation of opposite arms around the source axis: one arm holding the FAC, and the other arm holding a HPGe spectrometer (see schematic in Fig. 3), each of which is at a source-aperture distance of 50 cm. The equipment to power, control, and cool the x-ray sources, in addition to a well-chamber to provide a means of traceability, were kindly supplied by the Xoft Corporation, and all controls are outside the leaded-glass shielding maze that allows observation of the source and instruments during measurements.

**Fig. 4:**
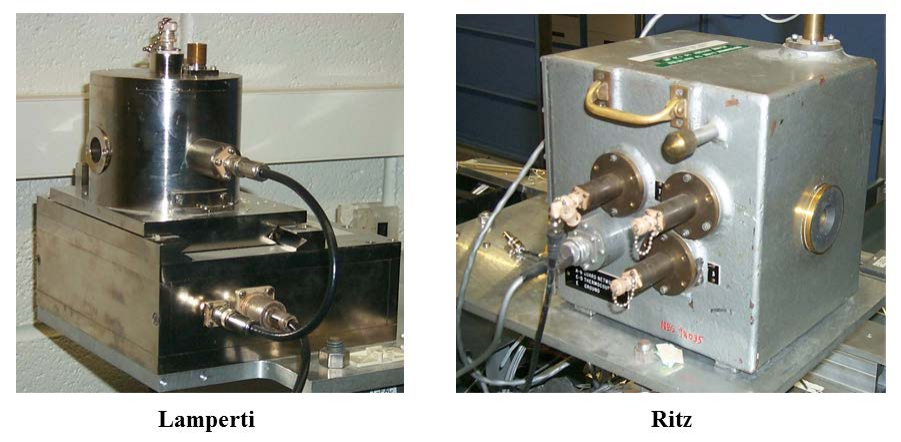
The NIST free-air ionization chambers used in the electronic brachytherapy facility.

The free-air ionization chamber that has been dedicated to this facility for the establishment and measurement of air-kerma is the Lamperti FAC [7]. The Ritz FAC [8] was used for comparison and verification purposes but is dedicated for use in the NIST Low Energy X-ray Calibration Range. Both FACs are proven national-standard instruments deemed suitable for the realization of air kerma for these sources. These FACs are shown in Fig. 4, and Table 1 lists their important parameters. The spectrometer is used to determine the true photon spectrum produced by the source and measured at the same distance as the FAC measurement. Spectrum measurements, as described later, are important to determine correction factors for the FACs.

**Fig. 5:**
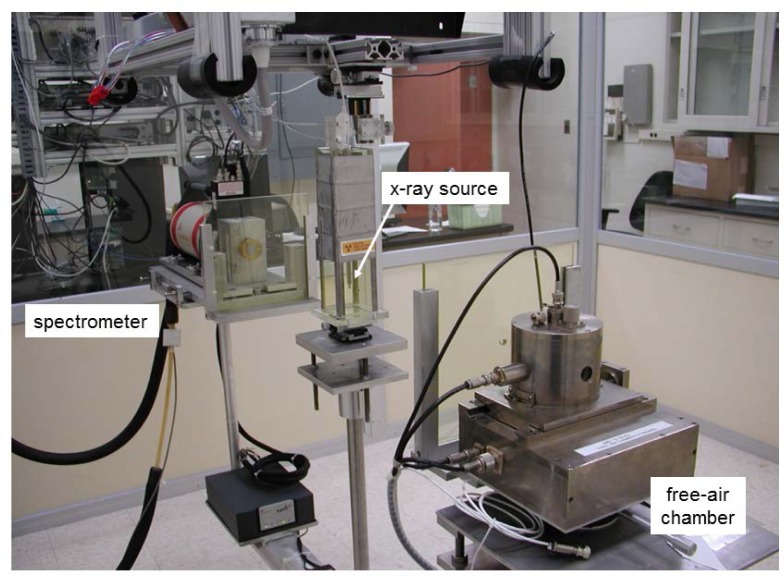
Measurement set-up with the Lamperti FAC.

**Fig. 6:**
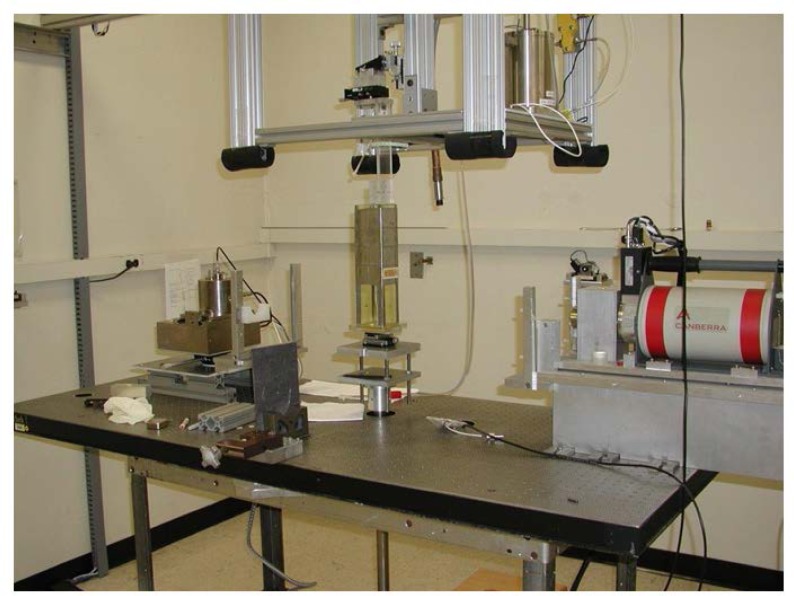
Amended measurement set-up with the Lamperti FAC.

**Fig. 7:**
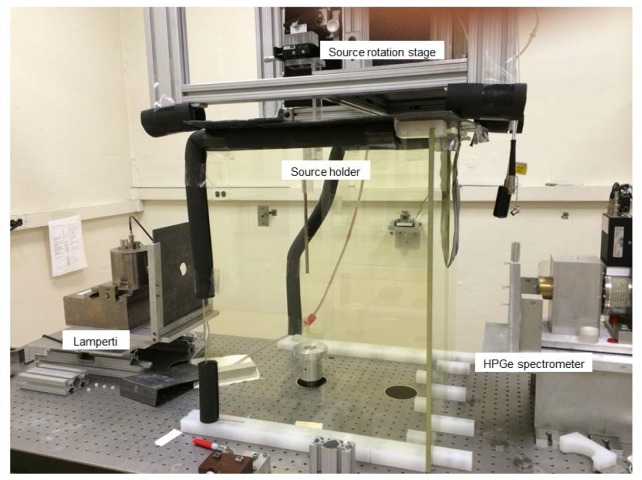
Low-scatter measurement set-up with the Lamperti FAC.

**Fig. 8:**
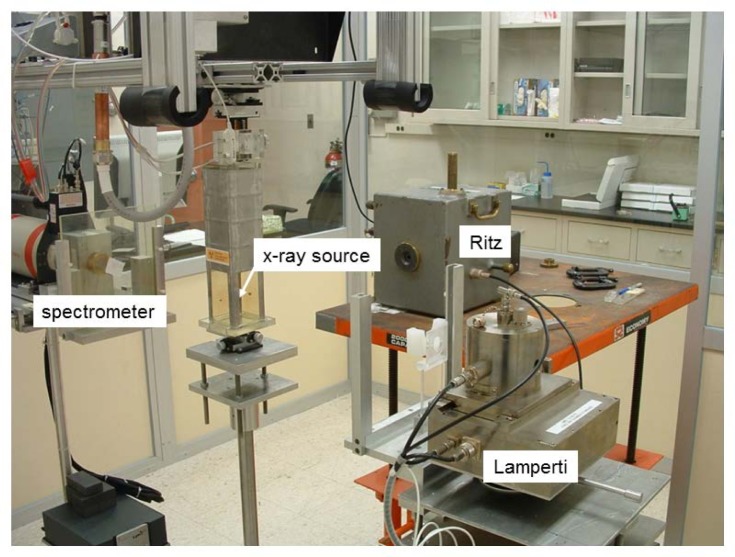
Set-up with the Ritz FAC in the measurement position. This arrangement does not show the source-rotation stage that was later added.

The original measurement set-up is shown in Fig. 5 for the Lamperti FAC. Because of some flexing of the arms that exacerbated alignment and reproducibility problems, the carousel arrangement was abandoned, the measuring-device platform fixed, and the source holder re-configured to allow rotation of the source. Figure 6 shows the amended geometric setup, which incorporates fixed positions for the FAC and HPGe detector and the rotation of the source inside the leaded glass source shield. Figure 6 also shows the well chamber located above the instrument platform, which is placed accordingly so that the limited-length high-voltage cable allows the source to easily be used for both the FAC measurements and the well-chamber measurements. The setup was later further amended by removing the small, lead-glass source shield and instead building a larger lead-glass surround. Because the rotation of the detectors around the source was eliminated, a rotation stage for the source was added. The final setup is shown in Fig. 7 and permits measurements with the least impact from scattered radiation. Figure 8 shows the Ritz FAC in the measurement position used for the FAC comparison.

## 3. Relevant Quantities

The quantity kerma (an acronym for kinetic energy released per unit mass), *K*, characterizes a beam of photons or neutrons in terms of the energy transferred to any material. Kerma is defined [9] as the quotient of d*E*_tr_ by d*m*, where d*E*_tr_ is the sum of the initial kinetic energies of all the charged particles liberated by uncharged particles (in our case, photons) in a mass d*m* of material. Thus,

$$K=\frac{dE_{\mathrm{tr}}}{dm}.$$ (1)

In the International System of Units (SI) [10] kerma has units of J kg^−1^; the special name for this unit is gray (Gy). Kerma rate, $\dot{K}$, is the quotient of d*K* by d*t*, where d*K* is the increment of kerma in the time interval d*t*. Our interest is in air kerma, *K*_air_, where d*m* is the mass of air.

Technically, air kerma, *K*_air_, is realized through a measurement of the related quantity exposure. Exposure, *X*, is the quotient of d*q* by d*m*, where d*q* is the absolute value of the mean total charge of the ions of one sign produced when all the electrons and positrons liberated or created by photons incident on a mass d*m* of dry air are completely stopped in dry air, thus

$$X=\frac{dq}{dm}.$$ (2)

The SI unit of exposure is C kg^−1^ (however, the older unit of Roentgen (R) is still used by some, where 1R = 2.58 × 10^−4^ C kg^−1^). The ionization produced by electrons emitted in atomic/molecular relaxation processes is included in d*q*. The ionization due to photons emitted by radiative processes (*i.e.*, bremsstrahlung and fluorescence photons) is not to be included in d*q*. Except for this difference, significant at high energies, the exposure, as defined above, is the ionization analogue of the dry-air kerma.

The quantities exposure and air kerma can be related through use of the mean energy expended in a gas per ion pair formed, divided by the elementary charge, *W*/e, where *W* is the mean energy expended in air per ion pair formed when the initial kinetic energy of a charged particle is completely dissipated in the air, and e is the elementary charge. Then

$$K_{\mathrm{air}}\approx X\cdot (W/e)/(1-\bar{g}).$$ (3)

The quantity *g* is the fraction of the kinetic energy of electrons (and positrons) liberated by the photons that is lost in radiative processes (mainly bremsstrahlung) in air. In Eq. (3), $\bar{g}$ is the mean value of *g* averaged over the distribution of the air kerma with respect to electron energy. The value of *W*/e for dry air currently adopted by the international measurement system is (33.97 ± 0.05) J/C [11], where the uncertainty pertains to one standard deviation.

It should be noted that the approximate equality in Eq. (3) is used here to reflect the fact that exposure includes the charge of electrons (or ions) liberated by the incident photons whereas *W* pertains only to the charge produced during the slowing down of these electrons. Thus the initial charge created by the interaction of the photons should, in principle, be discounted when transferring the exposure measurement for the determination of air kerma. This difference, although relatively small, tends to become more significant as the photon energy decreases. Additionally, *W* is not constant as perhaps implied in Eq. (3), but is known to increase at low energies [12]. At energies for which the variation of *W* with energy becomes important, one should consider also the effect of this increase.

It should be further noted that *K*_air_ is a point quantity corresponding in our measurement to a point at the center of the area of the aperture in its defining plane (see Ref. [6]). Alternatively, one can evaluate *K*_air_ in terms of only x-ray field quantities. For a fluence, *Φ*, of photons of energy *E*, the air kerma, *K*_air_, is given by [9]

$$K_{\mathrm{air}}=\int \Phi (E)E{\left(\frac{\mu_{\mathrm{tr}}}{\rho }\right)}_{\mathrm{air}}dE,$$ (4)

where *μ*_tr_/*ρ* is the mass energy-transfer coefficient in this case of air for photons of energy *E*, and *Φ*(*E*) is the distribution with respect to energy *E* of the photon fluence (*i.e.*, the quotient of d*N* by d*a*, where d*N* is the number of particles incident on a sphere of cross-sectional area d*a*). The coefficient *μ*_tr_/*ρ* is described in some detail in Refs. [13] and [14].

As recommended by the AAPM for the dosimetry of interstitial brachytherapy sources, the air-kerma strength, *S*_K_, is defined [15] as the product of the air-kerma *rate* at a point in free space (*vacuo*) located in the transverse bisecting plane at a distance *d* from the center (*i.e.*, cylindrical axis) of the source, and the square of the distance *d*. Thus,

$$S_{K}={\dot{K}}_{\mathrm{air}}^{\mathrm{vacuo}}(d)\cdot d^{2}.$$ (5)

The calibration distance *d* should be large enough that the source can be treated as a mathematical point. SI units of air-kerma strength are Gy m^2^ s^−1^; units appropriate for the traditional interstitial brachytherapy sources are μGy m^2^ h^−1^, which has been given the special symbol U by the AAPM. The quantity air-kerma strength is used in North America; the corresponding quantity used internationally is the reference air-kerma rate *in vacuo*, at a specified reference calibration distance, with units of μGy h^−1^. The reference calibration distance is usually specified as 1 m, in which case the air-kerma strength and the reference air-kerma rate would have the same numerical value, although formally with different units.

For the realization of air kerma, the results of a FAC measurement for x-ray beams are analyzed according to the measurement equation

$$K_{\mathrm{air}}=(W/e)\frac{Q_{\mathrm{net}}}{\rho_{{}_{\mathrm{air}}}V_{\mathrm{eff}}(1-\bar{g})}\prod_{i}k_{i},$$ (6)

where *Q*_net_ is the measured net ion charge (of one sign, corrected for cosmic-ray and system-generated background), *ρ*_air_ is the density of the air, and *V*_eff_ is the product of the aperture area and the length of the collecting volume. The air-kerma rate, ${\dot{K}}_{\mathrm{air}}$, is similarly obtained from Eq. (6), but with *Q*_net_ replaced by *I*_net_, the measured net ion current (of one sign, corrected for cosmic-ray and system-generated background). Equation (6) is an elaboration of combining Eqs. (2) and (3) in which the mass has been replaced by the product *ρ*_air_ *V*_eff_ and the application of the various necessary corrections is shown. See Refs. [3] and [4] for detailed descriptions of the FACs and the evaluation of the of correction factors, including the historical methods some of which the present report replaces.

In Eq. (6), *ρ*_air_ is determined from the measured temperature *T* and pressure *P* using the ideal gas law based on the density of dry air at standard conditions (0 °C, 1 atm or 101.325 kPa) or *ρ*_0_ = 1.2929 kg/m^3^; thus

$$\rho_{{}_{\mathrm{air}}}=\rho_{0}\frac{P}{101.325\;\mathrm{kPa}}\;\frac{273.15{}^{\circ}C}{273.15{}^{\circ}C+T}.$$ (7)

The effect of humidity is considered separately. The uncertainty in the determination of *ρ*_air_ is derived from the uncertainties of the measured values of *P* and *T*. The radiative-loss correction $\bar{g}$ is very small, effectively zero, for these x-ray beams, and will be discussed further below. The *k_i_* are correction factors for the effects of ion recombination, humidity, air attenuation, electron loss, photon scatter, fluorescence reabsorption, bremsstrahlung reabsorption, and the initial-ion production (mentioned above) within the FAC, and for the effects of scatter from the defining diaphragm. Except for the pressure-temperature correction, the correction factors in principle depend on the incident x-ray spectrum. Of these, except for the humidity and the initial-ion corrections, they depend also on the internal geometry of the FAC.

## 4. Source Spectrometry

The spectrometer consists of a Canberra liquid-nitrogen-cooled Ultra Low-Energy HPGe (GUL0110) detector coupled to their Lynx digital-signal-processing multi-channel analyzer (with their transistor-reset pre-amplifier). The GUL0100 detector incorporates an HPGe crystal with an active diameter of 11.3 mm (area of 100 mm^2^) and a thickness of 10 mm, behind a 0.025 mm Be cryostat window. About 34 pulse-height distributions were measured for nine S700 sources supplied by Xoft, all running at a tube potential of 50 kV and an anode current of 300 μA (the recommended parameters for clinical applications of these sources), except for two spectra for which currents of 50 μA and 30 μA were also used. The energy calibration of the spectrometer system was done using check sources incorporating ^57^Co (mainly using the 122.1 keV and 14.4 keV gamma rays) and ^109^Cd (mainly using the 22 keV Ag Kα x rays). The high-atomic-number pinhole aperture centered outside the Be window of the detector reduced count rates to a manageable level. This restricts incident photons to be incident on a small area at the center of the HPGe crystal, and proper alignment insures that the photons are incident normally, both conditions on which our knowledge of the detector-response functions is based. In principle, one can do reasonably accurate absolute dosimetry if the area of the pinhole is known, *i.e.*, one can measure the properly normalized photon fluence rate rather accurately. However, because of difficulties in alignment and the use of partial blocking of the pinhole in order to reduce the dead time of the spectrum measurement, only relative measurements were made (*i.e.*, the *shape* of the fluence spectrum), which is sufficient for our purposes.

The pulse-height distribution is the convolution of the incident photon energy spectrum and the detector-response function, which is a function of incident energy. The detector-response function itself is the convolution of the spectrum of energy deposited in the detector volume with the intrinsic resolution function for the detector (a result of the statistics of charge creation and collection). The systematics of the spectrum of energy deposited in pure Ge cylinders by photons incident at the center of a plane face was studied by Seltzer [16] using Monte Carlo radiation-transport calculations, and the results were schematized through a single-scattering approach to allow determination of the energy-deposition spectra for any practical-size Ge cylinder. This scheme, but using updated standard-reference-data photon-interaction cross sections for Ge [17], was used to generate energy-deposition spectra^2^ for the cylindrical crystal in the HPGe detector used in the measurements. The intrinsic resolution function for such a HPGe detector is very narrowly peaked (~500 eV FWHM for energies of interest here), which is their main advantage, so that numerous rather narrow energy bins (about 80 eV in our measurements) are used to record the pulse-height distribution. In such cases, it is impractical to deconvolve the pulse-height distribution through usual matrix-inversion techniques, so instead a simple backward-stripping algorithm [16] has been implemented in which a triangular array of energy-deposition spectra, using the same bins as the pulse-height distribution, are pre-calculated for photons incident with energies corresponding to the middle of the energy bins. The energy-deposition spectra are characterized by a delta function at the incident photon energy (corresponding to a total-energy deposition event, the so-called photopeak) with a probability that would give the photopeak area if the intrinsic resolution function were to be applied. Starting at about 60 keV, the photopeak delta function is normalized to the pulse-height distribution in that bin, and the thus normalized energy-deposition spectrum is subtracted from all the lower-energy bins; this is repeated for the next lower energy pulse-height-distribution energy bin, *etc*. The bin-by-bin normalizations then form the “true” incident photon spectrum, but still convolved with the intrinsic resolution function. The resolution function is so narrow it does not significantly distort our integrations of the spectrum over other functions of interest to obtain the needed results, although removing it and rendering the resultant x-ray peaks as delta functions would be more aesthetically pleasing.

**Fig. 9:**
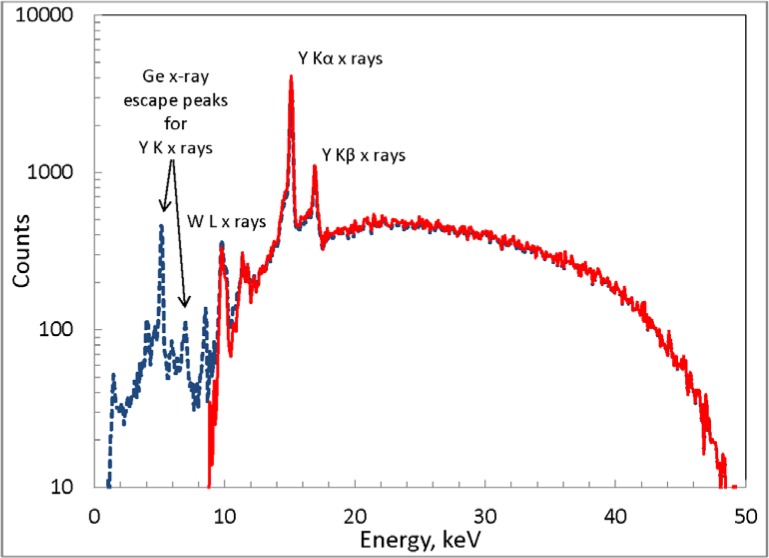
Measured pulse-height distribution (dashed blue curve) and derived true photon spectrum (solid red curve) for the Xoft Axxent source 903223 at 50 kV. Prominent characteristic x-ray peaks in the spectra are indicated, as are artifacts in the pulse-height distribution due to Ge x-ray escape peaks.

**Fig. 10:**
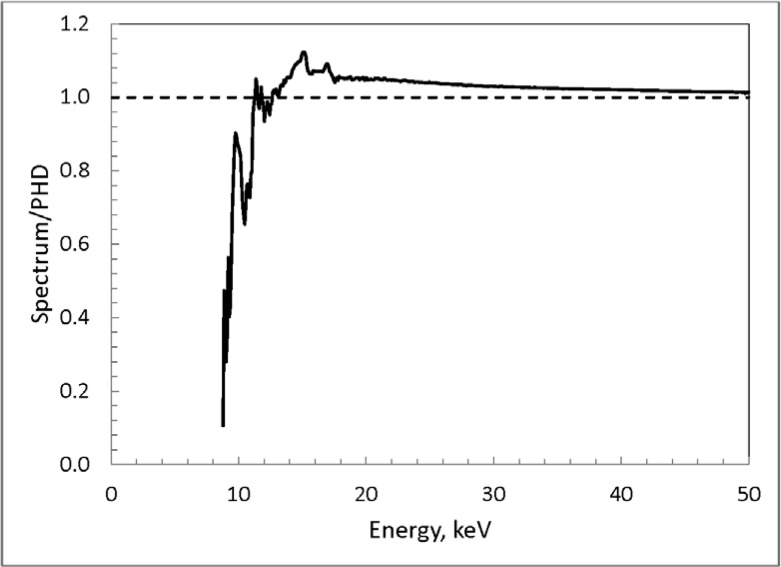
Ratio of the derived true photon spectrum to the measured pulse-height distribution for the Xoft Axxent source 903223 at 50 kV.

Illustrative results of this stripping method are shown in Fig. 9, from which a number of qualitative points can be noted. Prominent peaks in the spectrum at about 15 keV and 17 keV are due to characteristic x rays from yttrium emitted by the source (W L x-ray peaks are also seen at about 10 keV and 12 keV). The energy-deposition spectra include Ge x-ray escape peaks (total photoelectric absorption minus the escape of Ge characteristic x rays), so these peaks that are repeated at lower energies in the pulse-height distribution are removed. Additionally, the energy-deposition spectra include a continuum due to Compton scattering of the incident photon in the crystal and the subsequent escape of the scattered photon, which becomes more significant for the higher-energy incident photons. Thus the continuum below about from 6 keV to 8 keV in the pulse-height distribution is also effectively removed. This is perhaps better illustrated in Fig. 10, which gives the ratio of the “true” spectrum to the original pulse-height distribution. Down to about 15 keV, the ratio mainly reflects the inverse of the photopeak efficiency, with the removal of the Compton tails more noticeable at the lower energies.

For the nearly three dozen pulse-height distributions analyzed, the dead times recorded by the spectrometer system ranged from about 0.5 % to 5 % for two-thirds of the spectra and from 10 % to nearly 35 % for the other third. These dead times reflect the fraction of the time lost by the system because photons come faster than the time required to detect and process pulses. For large dead times, pulse pile-up is increasingly evident at energies above the spectrum end-point (50 keV). Unfortunately, inaccurate pulse processing and pile-up affect also the spectrum below the end-point, the effects of which are not easily accounted for in our system. Attempts were made to simulate such effects, but our lack of full understanding of the electronics and logic of the circuits led us to instead adopt a simple (but no doubt inaccurate) approach in which a straight-line “background” is assumed to stretch from the value of the stripped “true” spectrum at 50 keV down to that at about 7 keV^3^. Subtracting this from the stripped spectrum produces a modified spectrum cleanly extending from (about) 7 keV up to 50 keV; such a low-energy cut-off is consistent with the construction and energetics of the source (see Ref. [18]).

Although our final spectra share the same basic features and shape, they are not completely identical with one another. For example, the mean energy from our collection of spectra ranges from 23.3 keV to 27.9 keV, with an average mean energy of 25.3 keV with a standard deviation of 1.1 keV. There is no discernible correlation of mean energy with dead time, so the variations among the measurements could be due to real differences in the source output or to differences in alignment of the measurement that result in sampling somewhat different areas of the source. Fortunately, the functions over which we integrate to determine our correction factors are relatively insensitive to these differences.

## 5. FAC Correction Factors

Two of the correction factors (for ion recombination and for air attenuation within the FAC) are best, and traditionally, determined experimentally. The others can be determined computationally with good accuracy.

### 5.1 Ion Recombination, *k*_ion_

The recombination of ions with electrons before they are collected by the FAC is determined by standard means that involve varying the potential difference between the high-voltage and collecting plates of the FAC and the appropriate extrapolation to infinite field strength (see Ref. [19] for which recombination is assumed to be zero. As ion recombination depends on geometry and on the ionization rate, *k*_ion_ has been determined for appropriate air-kerma rates for each FAC. The results have been parameterized [3, 4] as

kion=0.9996+0.004573[K˙(Gy/s)]0.5for the Lamperti FAC,kion=1+0.087136K˙/(Gy/s)for the Ritz FAC. (8)

The typical air-kerma rates of the Axxent sources result in an ion recombination correction of unity for the Ritz FAC. An attempt was made to directly measure the ion recombination correction for the Lamperti FAC for rates typical of the Axxent sources. Because long-term stability could not be maintained for the sources, a reference radiation generated by a Rh anode with a similar rate and half-value layer was used. The Lamperti FAC was mounted in the NIST mammography range and the standard beam Rh/Rh40 (Rh anode, 0.029 mm Rh filtration, 40 kV tube potential) with a half-value layer (HVL) of 0.559 mm Al was chosen, as it is close to the HVL of the electronic brachytherapy sources. The tube current for the mammography unit was set so as to produce an air-kerma rate similar to that of the Axxent source. The result was a value for *k*_ion_ of 0.9994 compared to 0.9997 obtained from Eq. (8). This difference is not deemed significant, but rather is taken as an estimate of uncertainty, and because the results are in any case quite close to unity it was decided to set *k*_ion_ = 1 for this application. The ion recombination correction was determined by the Boag [20] two-voltage technique. If the rates of the Axxent sources change for future measurements, this correction can be re-determined.

### 5.2 Humidity, *k*_humidity_

The NIST reports air kerma for dry air. Humidity affects the results of the free-air chamber measurements in a number of ways. In principle, the photon attenuation coefficients for moist air are different from those for dry air. However, over the range of conditions pertinent to NIST measurements, the effect on the air-attenuation correction factor appears to be negligible. Depending on the water-vapor content, there can be small changes in the photon mass energy-absorption coefficient for air, the density of the air, and the *W*/e value for air. For the combined effects of these small changes, the humidity correction factor has been calculated (see Refs. [12] and [6]) as

$$k_{\mathrm{humidity}}=\frac{\rho_{\mathrm{dry}-\mathrm{air}}}{\rho_{\mathrm{humid}-\mathrm{air}}}\frac{W_{\mathrm{humid}-\mathrm{air}}}{W_{\mathrm{dry}-\mathrm{air}}}\frac{\int S(E)E{\left(\frac{\mu_{\mathrm{tr}}(E)}{\rho }\right)}_{\mathrm{dry}-\mathrm{air}}dE}{\int S(E)E{\left(\frac{\mu_{\mathrm{tr}}(E)}{\rho }\right)}_{\mathrm{humid}-\mathrm{air}}dE}.$$ (9)

The density of humid air was calculated using the equation of Giacomo [21], which takes into account the small CO_2_ content, the compressibility of the air-water-vapor mixture, and the enhancement factor (that expresses the fact that the effective saturation vapor pressure of water in air is greater than the saturation vapor pressure of pure vapor phase over a plane of pure liquid water). The variation of *W*_humid−air_/*W*_dry−air_ as a function of the partial pressure of water vapor was taken from the curve in Ref. [12] based on the results of Niatel [22]. Generally, the result for *k*_humidity_ is thus a complex function of temperature, pressure, relative humidity, and photon spectrum.

**Fig. 11:**
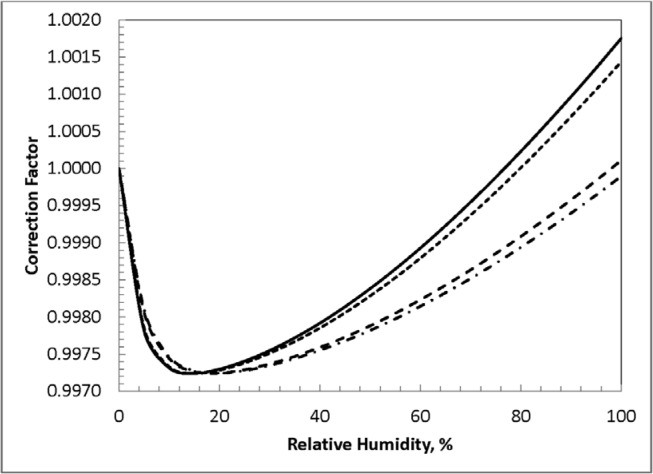
Humidity correction factors for the Xoft Axxent source at 50 kV. The solid curve is for a temperature of 24 °C and a pressure of 740 mm Hg (98.659 kPa); the short-dash curve is for 24 °C and 770 mm Hg (102.658 kPa); the long-dash curve is for 20 °C and 740 mm Hg (98.659 kPa); and the dot-dash curve is for 20 °C and 770 mm Hg (102.658 kPa).

The correction factor as a function of relative humidity, for temperatures of 21 °C and 24 °C and for pressures of 98.66 kPa (740 mm Hg) and 102.66 kPa (770 mm Hg), are shown in Fig. 11 for a spectrum typical of those measured. The temperatures and pressures chosen for these graphs have been judged to cover the measurement environment encountered in the NIST laboratory. The relative humidity in the laboratory (for which only an imprecise measurement is made) usually can vary from ≈15 % to ≈55 %. Considering the restricted range of values for these limits, it was deemed sufficient to simply use a mean value and to consider deviations as an uncertainty. The mean value is 0.9979, with an estimated standard uncertainty of 0.0004. This value, essentially 0.998, is the same as the humidity correction used for NIST free-air-chamber measurements of air kerma from our standard x-ray beams and from the sealed, low-energy-photon-emitting brachytherapy sources [6].

### 5.3 Air Attenuation, *k*_att_

The attenuation correction accounts for the attenuation of the beam from the aperture plane through the collecting volume in the FAC. The distance from the aperture plane to the mid-point of the collecting volume is termed the attenuation (or air-absorption) length for the FAC. The standard experimental method is to vary the attenuation length, keeping the aperture plane at the fixed source-to-FAC distance, and thus to derive the effective attenuation coefficient for the air kerma. One can also evaluate the attenuation from the incident spectrum using standard-reference photon-interaction data, but the result suffers from significant uncertainties due to inherent uncertainties of the photon-interaction data and of the role of scattering in the chamber. The attenuation correction factors and the effective attenuation coefficients for the FACs were evaluated from the spectra according to

$$k_{\mathrm{att}}=\frac{\int S(E)E\frac{\mu_{\mathrm{tr}}(E)}{\rho }dE}{\int S(E)\exp[-\mu (E)d_{\text{offset}}]\frac{\left\{1-\exp[-\mu (E)d_{\text{collector}}]\right\}}{\mu (E)d_{\text{collector}}}E\frac{\mu_{tr}(E)}{\rho }dE},$$ (10)

where *S*(*E*) is the photon spectrum (unnormalized fluence spectrum), *μ*(*E*) is the photon attenuation coefficient for air for a photon of energy *E*, *d*_offset_ is the distance from the aperture plane to the edge of the collector plate, and *d*_collector_ is the length of the collector plate along the beam axis (*i.e.*, the attenuation length is *L* = *d*_offset_ + *d*_collector_/2).

Photon mass attenuation and mass energy-transfer coefficients for dry air were initially taken from the XCOM database [17]. These are based on the direct results of Scofield’s [23] relativistic calculations of the photoelectric-absorption cross section for single bound electrons moving in a Hartree-Slater central potential. The work of Pratt and colleagues [24–27] shows that well above the electron binding energy the cross section is proportional to the bound-state wave function near the origin, and recommends the renormalization of results from limited-accuracy atomic models by the square of the ratio of the near-origin wave function from a more accurate atomic model to that from the model used in the calculation. Thus Scofield [23] listed renormalization factors to convert from Hartree-Slater to Hartree-Fock results (for all sub shells) for atomic numbers *Z* = 1 − 101. The renormalized cross sections were used in the NBS (now NIST) databases [28, 29, 13] up until 1986 when the reviews by Saloman and Hubbell [30, 31] indicated that, on the whole, agreement with experiment is better if the renormalization is not done. Thus, the unrenormalized Scofield photoelectric absorption cross sections are currently used in XCOM. Recent measurements with synchrotron radiation from 10 keV to 60 keV [32, 33] for air, however, seem to favor the renormalized results. Therefore, evaluations were done using mass attenuation and mass energy-transfer coefficients based on both unrenormalized and renormalized photoelectric-absorption cross sections.

The effective attenuation coefficient was then estimated according to

$${\left(\frac{\mu }{\rho }\right)}_{\mathrm{eff}}=\frac{\ln(k_{\mathrm{att}})}{d_{\text{offset}}+d_{\text{collector}}/2}.$$ (11)

The results of such analyses of the 34 spectra give values of *k*_att_ that vary from 1.0064 to 1.0110 for the Lamperti FAC and from 1.0208 to 1.0360 for the Ritz FAC, and values of (*μ*/*ρ*)_eff_ that vary from 1.36 cm^2^/g to 2.35 cm^2^/g for the Lamperti FAC and from 1.35 cm^2^/g to 2.32 cm^2^/g for the Ritz FAC. Mean values and their standard deviations are given in Table 2.

Measurements of the effective attenuation coefficient were reported by Davis [18] in his extensive study of the dosimetry of these sources. Davis used the UW Attix free-air chamber [34], with an attenuation length, *L*, of 19 cm. He reports values of the attenuation-correction factors for a number of sources for the UW Attix chamber from 1.035 to 1.045, with a typical value of 1.041. Applying Eq. (11), one obtains values of (*μ*/*ρ*)eff of from 1.513 cm2/g to 1.936 cm^2^/g. Because the spectra are not monoenergetic, there is some beam hardening over the attenuation lengths in such FACs; however, one can interpolate our calculated results for the NIST Lamperti (*L* = 3.9 cm), the Ritz (*L* = 12.7 cm), and the Wyckoff-Attix (*L* = 30.8 cm) FACs, with a result (using renormalized photoelectric-absorption cross sections) for *L* = 19 cm estimated to be 1.82 cm^2^/g, in good agreement with Davis’ result. The NIST Attix free-air ionization chamber [4] was also used to directly measure the air-attenuation corrections for various sources. These data were used to determine the corrections for the Lamperti and the Ritz FACs; the results approximate those listed in Table 2, with an average value of 1.0083 for the Lamperti chamber and 1.0268 for the Ritz chamber.

Although the Lamperti FAC is relatively insensitive to variations of (*μ*/*ρ*)_eff_, differences among source spectra and air density during measurements recommend that the attenuation-correction factor be determined for each source. This is difficult due to the variability of the geometric setup and the alignment of the required lead shield. Therefore the routine is to measure the air-attenuation correction to verify that it approximates the value in Table 2, which is then used for the calculation of air kerma.

### 5.4 In-Chamber Radiation-Transport Corrections

These correction factors have been evaluated using data from Monte Carlo photon-transport calculations done for the Lamperti FAC and Ritz FAC geometries by Burns^4^ and whose implementation is described in somewhat more detail by Seltzer [35]. These correction factors are defined in terms of ratios of relevant functions derived from the Monte Carlo calculations. The functions have been determined for photon energies spanning the energies for which the FACs were designed. Each correction factor is evaluated by integrating separately the appropriate function(s) in the numerator and the function(s) in the denominator before forming the ratio (see Ref. [35]).

As mentioned earlier, we are fortunate that the ratios are relatively insensitive to the changes in the spectrum, which is particularly true for these correction factors. In addition to uncertainties associated with the source spectrum, there are also uncertainties associated with the basis functions derived from the Monte Carlo calculations, which are described in Ref. [35].

#### Electron-loss correction, *k*_el_

Energetic electrons can leave (and enter) the collection volume, with only a portion of their energy expended in ionization being collected. The collecting volume is defined by the area of the collecting plate and the electrode separation. The results for the electron-loss correction factors are given in Table 3.

#### Photon-scatter correction, *k*_sc_

Ionization produced by electrons resulting from photons scattered out of the aperture-defined beam is not included in the definitions of exposure and of air kerma. The results for the photon-scatter correction factors are given in Table 4.

#### Fluorescence-reabsorption correction, *k*_fl_

The ionization collected due to re-absorption of fluorescence photons is not included in the definitions of exposure and of air kerma. The results for the photon-scatter correction factors are given in Table 5.

#### Bremsstrahlung-reabsorption correction, *k*_br_

The ionization collected due to re-absorption of bremsstrahlung photons is not included in the definitions of exposure and of air kerma. This is associated with but separate from the $\left(1-\bar{g}\right)$ correction in Eq. (6). The NIST currently assumes $\bar{g}$ in Eq. (6) is identically zero for its calibration x-ray beams. If the appropriate small values were to be used, then the use of *k*_br_ would correct for effects of re-absorption. Thus, the adopted result is instead an estimated uncertainty associated with the assumption that $k_{\mathrm{br}}/(1-\bar{g})=1$ for the Xoft Axxent sources operated at 50 kV, as given in Table 6.

### 5.5 Initial-Ion

The initial-ion-production correction factor, *k*_ii_, serves to discount the initial charge created by the interaction of the photons in the FAC that is not to be included in the realization of air kerma. The initialion correction has been considered by Büermann *et al.* [32], and by Takata and Begum [36]. This correction appears to be relatively small, *i.e.*, rather close to unity, but does become more significant, approaching about 0.995 for standard x-ray beams with the lowest mean energies (~ 7 keV). For the Xoft Axxent source at 50 kV, the mean energy of the beam from our measurements suggests a value of 0.9981 based on the data of Takata and Begum [36] or a value of 0.9978 using the procedures of Büermann *et al.* [32]. Thus, the value of 0.9980 might be a reasonable compromise, with an estimated standard uncertainty of 0.0004 suggested by the standard deviation for the measured mean energy of the beam and by the difference from the two analyses. However, the NIST has not included this correction for its x-ray standards, and its use is still being discussed in the international forum. Therefore the NIST will continue to assume a value of unity but now with an estimated uncertainty of 0.002 for the Xoft Axxent source.

### 5.6 Diaphragm Scatter, *k*_dia_

The diaphragm-scatter correction factor corrects for the effects of photons scattered from the diaphragm surfaces and transmitted through the defining edge of the diaphragm (and of fluorescence photons emerging from the diaphragm) due to the divergence of the x-ray beam entering the diaphragm. Recent evaluations of this correction have been done at a number of laboratories (see, *e.g.*, Refs. [37–40]), but because the results (derived from Monte Carlo radiation-transport calculations) depend on geometric details of the FAC they are difficult to transfer with precision to the NIST FACs.

However, because this correction is expected to be small for the spectrum considered here, it was felt sufficient to continue with the practice at the NIST to assume that *k*_dia_ = 1.0, but to assign a reasonable uncertainty. The various reports referenced above indicate a correction factor no smaller than 0.999 for diaphragm diameters of about 10 mm, similar to that in the NIST Ritz FAC. This is remarkably consistent with the 57-year-old estimate of Wyckoff and Attix [41] that diaphragm scatter is less than about 0.001 for a 10 mm diameter diaphragm and, presumably, a cylindrical thickness of about 10 mm (as in the Ritz FAC). Thus, an uncertainty associated with *k*_dia_ = 1.0 is estimated to be 0.001 for the Ritz FAC. As McEwan [42] has pointed out, the correction factor should vary as the inverse of the diaphragm diameter, *d*. More completely, the correction factor should vary as the ratio of the area of the cylindrical sides of the diaphragm to the defining area of the diaphragm, which is 4*h*/*d*, with *h* the thickness of the cylindrical diaphragm aperture. The value of *h* for the Lamperti FAC is 5 mm, giving roughly the same ratio as for the Ritz FAC. Although the correction factor no doubt depends also on the remaining geometric details, which are different in the Ritz and Lamperti FACs, to a first approximation the correction factor should be roughly the same and also rather close to unity. Thus it seems reasonable to assign the same uncertainty.

### 5.7 Correction to Reference Conditions

Although not a correction factor, the NIST states air kerma for reference conditions of an atmospheric pressure, *P*, of 101.325 kPa and temperature, *T*, of 22 °C. Thus, the result of the measurement is multiplied by $\frac{101.325\;\mathrm{kPa}}{P}\frac{273.15{}^{\circ}C+T}{295.15{}^{\circ}C}$.

## 6. Conversion of Air Kerma to Air-Kerma Strength

Equation (5) can be recast as

$$S_{K}={\dot{K}}_{\mathrm{air}}^{\mathrm{vacuo}}(d)\cdot d^{2}={\dot{K}}_{\delta }^{\mathrm{air}}(d)\left[\frac{{\dot{K}}_{\delta }^{\mathrm{vacuo}}(d)}{{\dot{K}}_{\delta }^{\mathrm{air}}(d)}\right]d^{2},$$ (12)

where a change in notation on the right of the second equal symbol assumes all kerma rates are air-kerma rates and the subscript *δ* indicates a low-energy cut-off for the air kerma. For low-photon-energy, sealed-source, radionuclide brachytherapy, the NIST uses a low-energy cut-off, *δ*, of 5 keV, consistent with the recommendations of the AAPM [43]. With an effective low-energy cut-off of 6 keV to 8 keV for the spectra of the Xoft Axxent x-ray sources, this value of *δ* is naturally maintained.

The square bracket in Eq. (12) can be evaluated from the true photon spectra measured for the Xoft Axxent sources. The spectra *in vacuo* are determined assuming simple attenuation of the photons by the 50 cm of air, and the air kerma in the numerator and the denominator is evaluated using Eq. (4). As was done in Sec. 5.3, evaluations were done using mass attenuation and mass energy-transfer coefficients based on both unrenormalized and renormalized photoelectric-absorption cross sections. The value obtained for the ratio in Eq. (12) is 1.12 with a relative standard deviation of 1.7 %, with a relative difference of only 0.3 % between the results using unrenormalized and renormalized photoelectric-absorption cross sections, due to substantial cancellations in the ratio.

The NIST determines *S*_K_ for sealed-radionuclide low-energy photon-emitting interstitial brachytherapy sources but not for its standard electrically generated x-ray beams. The Xoft Axxent sources can be considered to fall in between. Because there is no compelling reason to adopt air-kerma strength as the reporting quantity, the NIST chose to report reference air kerma at 50 cm in air so as to avoid the relatively large added uncertainty associated in this case with air-kerma strength. This choice can change depending on possible future developments in clinical-dosimetry protocols by the AAPM.

## 7. Summary

A summary of the adopted values of correction factors derived from the analysis outlined above is given in Table 7. The adopted uncertainties of the factors used in the determination of the air kerma are listed in Table 8.

## 8. Comparison of NIST National Standard Instruments

The free-air ionization chamber that has been dedicated for the measurement of air-kerma for electronic brachytherapy is the Lamperti FAC [7]. However, the Ritz FAC [8] was used for comparison and verification purposes although it is dedicated for use in the NIST Low Energy X-Ray Calibration Range. Both FACs are proven national-standard instruments, compared internationally [44], and deemed suitable for the realization of air kerma for these sources. The Lamperti FAC was selected due to its availability, energy range, and appropriate dimensions.

A series of comparisons between the Lamperti and Ritz chambers were made. The chambers were positioned 90 degrees from each other, both 50 cm from the source, through the use of a temporary support for the Ritz FAC (see Fig. 8). The source was rotated to eliminate any angular variation (initially the FAC’s were compared with the original rotating setup). Various FAC apertures were used for the Ritz chamber to verify the geometry; the Lamperti chamber accepts only one size aperture. The use of various diameter apertures in a parallel plate FAC is a method used to verify proper geometry and alignment. The calculated correction factors, listed in Table 7, provided the basis for this comparison. Initial agreement was reached at the 0.5 % level. However, once the fixed geometry was established, the FACs agreed to within 0.1 %. Such good agreement demonstrates that both chambers can be used to realize air-kerma for these electronic brachytherapy sources.

## 9. Well Chamber as Air-Kerma Transfer Instrument

The air-kerma rates from numerous S700 electronic brachytherapy sources were determined during January of 2013 and April of 2014. The NIST determined the air-kerma rate in terms of Gy/s for the sources at a distance of 50 cm using the Lamperti chamber. A well chamber provided by Axxent, a Standard Imaging Model HDR 1000 Plus with the Axxent source holder, resides in the NIST facility, see Fig. 6. It has been evaluated for use as a transfer instrument. The NIST calibration coefficients of the well chamber have units of Gy/(A s) normalized to reference conditions of 295.15 K and 101.325 kPa. It has been demonstrated that the well chamber is an appropriate transfer standard for the electronic brachytherapy sources, as well as an efficient means of determining the stability of the sources. Of the 26 sources that have been measured with the well chamber, the average standard deviation of the collected charge is 0.1 %. A typical expanded, combined uncertainty of the well chamber calibration coefficient is 0.75 % of which 0.7 % is assigned to the uncertainty in the air-kerma rate of the Axxent source.

## Figures and Tables

**Table 1:** Critical dimensions for the NIST parallel-plate free-air chambers. All dimensions are in units of cm.

	Lamperti (10 kV – 50 kV)	Ritz (20 kV – 100 kV)
Diaphragm diameter	0.5	1.0
Collector length	1.0	7.0
Collector width	5.0	9.0
Electrode separation	4.0	9.0
Attenuation length	3.9^a^	12.7

a The Lamperti FAC has been modified with new diaphragms so that the attenuation length is now 4.0 cm; however, this slight change has not been included in the evaluations of this report.

**Table 2:** Estimated calculated values of the attenuation corrections for the Lamperti and Ritz FACs for Xoft Axxent sources operated at 50 kV. These estimates are based on the use of renormalized photoelectric-absorption cross sections (see text) and an air density for reference conditions (*T* = 22 °C and *P* = 101.325 kPa). The uncertainties are the standard deviations of results from the analysis of 34 spectra.

	*k*_att_	Relative Standard Uncertainty, %	(*μ*/*ρ*)_eff_	Relative Standard Uncertainty, %
Lamperti FAC	1.0087	0.11	1.85	13.2
Ritz FAC	1.0283	0.36	1.83	13.1

**Table 3:** Adopted values of the electron-loss correction factors for the Lamperti and Ritz FACs for Xoft Axxent sources operated at 50 kV. The uncertainties are the standard deviations of results from the analysis of 34 spectra, combined with the standard uncertainties associated with the basis functions used to evaluate the correction factor.

	*k*_el_	Relative Standard Uncertainty, %
Lamperti FAC	1.0008	0.06
Ritz FAC	1.0000	0.08

**Table 4:** Adopted values of the photon-scatter correction factors for the Lamperti and Ritz FACs for Xoft Axxent sources operated at 50 kV. The uncertainties are the standard deviations of results from the analysis of 34 spectra, combined with the standard uncertainties associated with the basis functions used to evaluate the correction factor.

	*k*_sc_	Relative Standard Uncertainty, %
Lamperti FAC	0.9987	0.03
Ritz FAC	0.9970	0.03

**Table 5:** Adopted values of the fluorescence-reabsorption correction factors for the Lamperti and Ritz FACs for Xoft Axxent sources operated at 50 kV. The uncertainties are the standard deviations of results from the analysis of 34 spectra, combined with the standard uncertainties associated with the basis functions used to evaluate the correction factor.

	*k*_fl_	Relative Standard Uncertainty, %
Lamperti FAC	0.9979	0.05
Ritz FAC	0.9969	0.05

**Table 6:** Adopted values of the quotient of the bremsstrahlung-reabsorption correction factor by the radiative-loss correction factor for the Lamperti and Ritz FACs for Xoft Axxent sources operated at 50 kV. The uncertainties are the standard deviations of results from the analysis of 34 spectra, combined with the standard uncertainties associated with the basis functions used to evaluate the correction factor.

	$k_{\mathrm{br}}/(1-\bar{g})$	Relative Standard Uncertainty, %
Lamperti FAC	1.0000	0.02
Ritz FAC	1.0000	0.02

**Table 7:** Adopted correction factors for measurements made with the NIST FACs for the Xoft Axxent source at 50 kV.

Factor	For:	Lamperti	Ritz
1 *k*_ion_	ion recombination	≈1.0000	≈1.0000
2 *k*_humidity_	humidity of air	0.998	0.998
3 *k*_att_	Attenuation	1.0087	1.0283
4 *k*_el_	electron loss	1.0008	1.0000
5 *k*_sc_	photon scatter	0.9987	0.9970
6 *k*_fl_	fluorescence reabsorption	0.9979	0.9969
7 *k*_br_/(1−*g*)	effects of bremsstrahlung	1.0	1.0
8 *k*_ii_	initial ion	1.0	1.0
9 *k*_dia_	diaphragm scatter	1.0	1.0
П *k*_1−9_		1.0041	1.0200
*K*_vac_/*K*_air_	conversion to air-kerma strength	1.12	1.12

**Table 8:** Estimated relative standard uncertainties in the determination of air kerma for the Xoft Axxent source at 50 kV.

Component	For:	Relative standard uncertainty, %		
		Type A	Lamperti	Ritz
			Type B	Type B
*Q*_net_, *I*_net_	net charge or current	*s*_Q_^a^, *s*_I_^a^	0.06	0.06
*W*/e	mean energy per ion pair	–	0.15	0.15
*ρ*_0_	air density	–	0.03	0.03
*V*_eff_	effective volume	0.04	0.01	0.01
*k*_ion_	ion recombination	0.03		
*k*humidity	humidity of air		0.03	0.03
*k*_att_	attenuation	–	0.11	0.36
*k*_el_	electron loss	–	0.06	0.08
*k*_sc_	photon scatter	–	0.03	0.03
*k*_fl_	fluorescence reabsorption	–	0.05	0.05
*k*_br_/(1−*g*)	effects of bremsstrahlung	–	0.02	0.02
*k*_ii_	initial ion	–	0.04	0.04
*k*_dia_	diaphragm scatter	–	0.10	0.10
*k*_d_	electric field distortion	–	0.20	0.20
	aperture penetration	negligible		
	chamber face penetration	negligible		
	polarity difference	0.02		
combined	air kerma	0.054	0.316	0.469

a Determined as the standard deviation of the mean of the measurement.

## Footnotes

1 Brand names, model designations, and/or manufacturers are given for identification only, and do not imply recommendation or endorsement by the National Institute of Standards and Technology, nor does it imply that the products are necessarily the best available for the purpose.

2 These results ignore any effects of photons scattering from the walls of the cryostat back into the crystal.

3 The minimum energy was occasionally a bit higher (~8 keV) to avoid negative values for the spectrum produced by the stripping procedure.

4 Personal communication from D. T. Burns, 2001.
